# Development of Functional Phthalocyanine-Based Associate towards an Effective Fluorimetric Detection of Hg(II)

**DOI:** 10.3390/molecules23020418

**Published:** 2018-02-14

**Authors:** Guang-Xin Du, Tao Zhou, Meng-Lin Guo, Ping Huang, Ya-Bin Deng, Dong-Hui Li

**Affiliations:** Cancer Research Center, Medical College, Xiamen University, Xiamen 361102, China; 15071183472@sohu.com (G.-X.D.); zeltar@163.com (T.Z.); 24520131153557@stu.xmu.edu.cn (M.-L.G.); Huangp@xmu.edu.cn (P.H.); dyb@xmu.edu.cn (Y.-B.D.)

**Keywords:** phtalocyanine, fluorescence, mercury, probe

## Abstract

In acidic media, cationic phthalocyanine Alcian blue 8GX, has an efficient fluorescence quenching effect on anionic phthalocyanine tetrasulphoaluminium phthalocyanines (AlS_4_Pc), forming an almost non-fluorescent associate. Based on this discovery, a red-emitting fluorescent probe consisted of AlS_4_P_C_ and Alcian blue 8GX has been developed through molecular assembly. Further studies indicated that the presence of Hg(II) ion has a significant fluorescence recovery effect of the probe. Notably, only Hg(II) can significantly restore the fluorescence of AlS_4_Pc-Alcian blue 8GX system which was revealed from the screening experiments of common metal ions, which confirmed that the fluorescence recovery by other metal ions is very weak or even unrestored, showing high specificity and sensitivity AlS_4_Pc-Alcian blue 8GX to Hg(II). Thus, a new fluorimetry for Hg(II) with high specificity and high sensitivity in a wide concentration range has been established using AlS_4_P_c_-Alcian blue 8GX associate as a red-emitting fluorescent probe. It is more noteworthy that this study opens a new way for development and application of functional phthalocyanine based red-emitting fluorescent probes.

## 1. Introduction

Mercury and its compounds are highly toxic substances that can cause poisoning to the central nervous system, kidneys, mouth, and skin of humans. Mercury can enter the human body directly through the skin, digestive tract, or respiratory tract, and also through the strong enrichment in the food chain accumulated in the atmosphere as well as in soils, oceans, and other environments [1,2]. The inorganic Hg ions in the environment can be converted to more toxic organic Hg under certain conditions. Hg ions can bind to the hydrophobic part of proteins to inhibit the activity of enzymes and block the metabolism of cells, which leads to perception and behavioral disorders and nerve damage, and results in multiple system damages causing mainly neurotoxicity and nephrotoxicity to the body. Mercury not only damages the living environment, but also greatly threatens human health. Therefore, it is quite significant to develop a fast and simple technology to detect mercury ion [3,4,5].

Many methods have been reported for the determination of mercury, including atomic absorption spectrometry (AAS) [6,7], atomic fluorescence spectrometry (AFS) [8,9], atomic emission spectrometry (AES) [10], mass spectrometry [11,12], chromatography [13], and electrochemical methods [14,15]. However, these analytical methods are either require expensive instruments or have complicated operating procedures. Therefore, the development of cheap and simple techniques for the detection of mercury ion has become an important area of research in these days. Fluorescent technology is simple and easy to operate, and has the characteristics of high sensitivity, high selectivity, real-time, online, and in situ detection. It is widely used in the biochemical analysis [16,17], food analysis [18], pharmaceutical analysis [19], environmental analysis [20,21], and more attention is paid in the field of detection of Hg(II). In the last decade, it has been an attractive and impressive topic of development of organic small molecules based fluorescent probes for the analysis or imaging of Hg(II) [3,22,23]. These so-called “on-off” fluorescent probes focus on synthesis based on the parent structure of BOPDY [24], Rhodamine [25], and naphthalimide [26]. Though these newly emerged probes respond to Hg(II) with high specificity and sensitivity, there are still drawbacks as follows: most of the determination should be performed in organic or organic–aqueous media due to the poor solubility of these compounds in water; almost all of these derivatives are laboratory synthesized, and they are commercially unavailable, thus the applications are limited.

In this study, a phthalocyanine associate consisting of oppositely charged phthalocyanines—which are fully water-soluble and commercially available—has been constructed for fluorescent recognition of Hg(II). Tetrasulfoaluminum phthalocyanine (AlS_4_Pc) is a strong fluorescent compound emitted at the red wavelength range with good water solubility, stability, low photobleaching effect, and high fluorescence quantum yield [27], and thus its application in the field of fluorescence analysis has attracted intensive attention in recent years [28,29,30,31,32]. This study found that Alcian blue, a cationic copper phthalocyanine compound, has an efficient fluorescence quenching effect on the AlS_4_Pc to form an almost no fluorescence ion-pair associate (AlS_4_Pc-Alcian blue 8GX) in acidic or weak acidic medium, whereas the fluorescence recovered significantly in the presence of Hg(II). Interestingly, the screening experiments showed that only Hg(II) from the common metal ions can significantly restore the fluorescence of AlS_4_Pc-Alcian blue 8GX system, while the presence of other metal ions has a slight or no fluorescence recovery, indicating the highly specific responsiveness of Hg(II). Based on this phenomenon, a new method for quantitative analysis of Hg(II) with high specificity and high sensitivity has been established through the red fluorescent probe consisting of AlS_4_Pc-Alcian blue 8GX.

## 2. Results and Discussion

### 2.1. Molecular Structure of AlS_4_Pc and Alcian Blue 8GX

**Figure 1:**
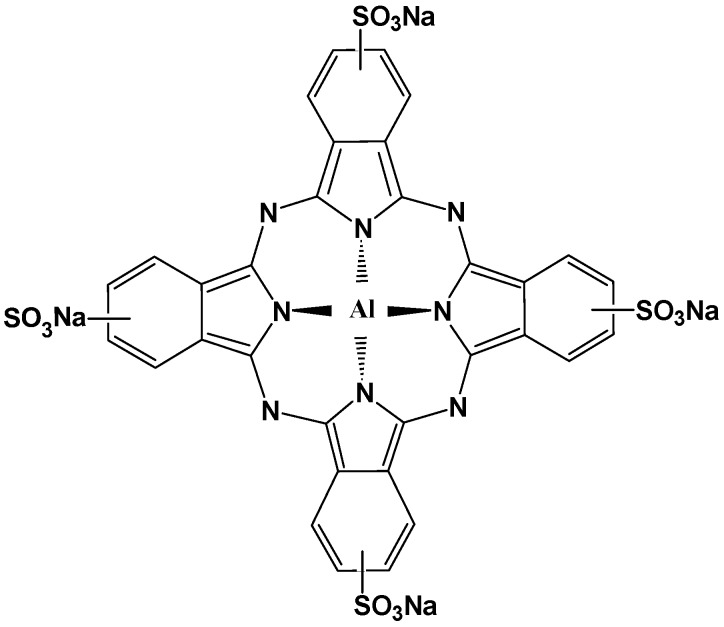
Molecular structure of AlS_4_Pc.

**Figure 2:**
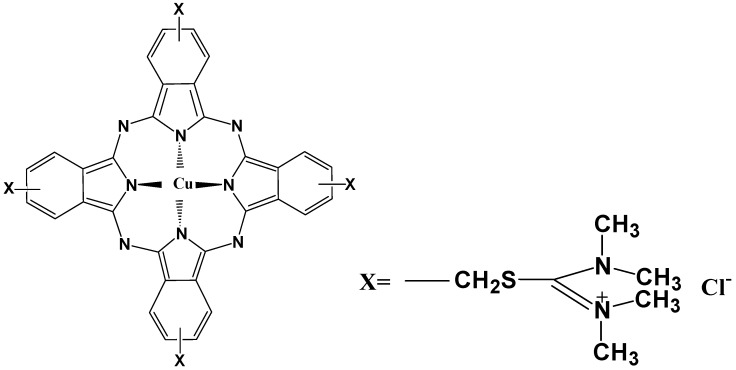
Molecular structure of Alcian blue 8GX.

Tetrasulfoaluminium phthalocyanine (AlS_4_Pc) is a strong fluorescent compound with a parent porphyrin-like structure as shown in Figure 1. Its strong polar and negatively-charged sulfonic acid groups on the four peripheral benzene rings greatly enhance its water solubility thereby facilitating its application in aqueous systems. Alcian blue 8GX (Figure 2) has the same parental structure as AlS_4_Pc, but it does not emit fluorescence because of its central coordinating atom which is a paramagnetic divalent copper ion, and each of the peripheral benzene rings is connected with sulfur-containing positively charged groups through thioether bond.

### 2.2. Fluorescence Properties of AlS_4_Pc

Tetrasulfoaluminium phthalocyanine (AlS_4_Pc) was found to be a fluorescent compound with high quantum yield and showed excellent photo-chemical stability [27]. With the attachment of sulphonic acid group on each of the peripheral benzenes, AlS_4_Pc shows large solubility in water phase. Considering these merits, AlS_4_Pc exhibits great potential as a novel red-emitting fluorescent probe. It has been used as a fluorescent probe and applied to quantitative analysis based on its distinct characteristics in previous works [28,29,31,32]. The fluorescence emission peak of AlS_4_Pc locates around 685 nm with two excitation bands; one in the short-wavelength region (called Soret band) and the other in the long-wavelength region (called Q band). As the shortwave excitation will generate secondary scattering strongly around 700 nm (near the fluorescence emission peak), the excitation wavelength is selected in this study at long wavelengths (around 620 nm) to avoid interference from scattering light.

### 2.3. Spectral Behavior and the Mechanism of the Reaction System

**Figure 3:**
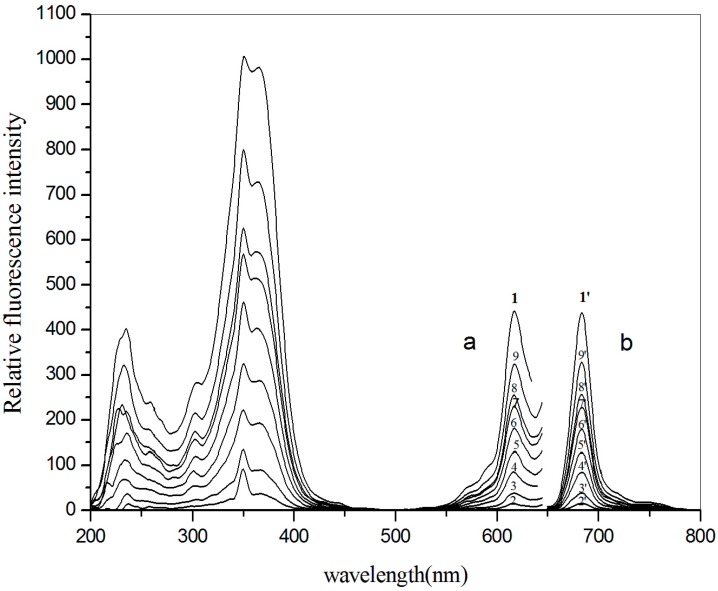
Excitation (**a**) and emission (**b**) of AlS_4_P_C_-Alcian blue 8GX in the presence of Hg(II) in an aqueous medium with a pH of 5.0. 1-1’. Buffer + AlS_4_Pc, [AlS_4_Pc] = 1.0 × 10^−6^ mol/L, [EDTANa_2_] = 1.0 × 10^−4^ mol/L. 2-2’. Buffer + AlS_4_Pc + Alcian blue 8GX, [AlS_4_Pc] = 1.0 × 10^−6^ mol/L, [Alcian blue 8GX] = 1.50 × 10^−6^ mol/L, [EDTANa_2_] = 1.0 × 10^−4^ mol/L; 3-3’, 4-4’, 5-5’, 6-6’, 7-7’, 8-8’, 9-9’. Buffer + AlS_4_Pc + Alcian blue 8GX + Hg(II), [AlS_4_Pc] = 1.0 × 10^−6^ mol/L, [Alcian blue 8GX] = 1.50 × 10^−6^ mol/L, [EDTANa_2_] = 1.0 × 10^−4^ mol/L, concentrations of Hg(II) for curves 3–9 and 3’–9’: 0.2, 0.4, 2.0, 10.0, 50.0, 100.0 and 200.0 µmol/L, respectively.

In this study, the fluorescence of AlS_4_Pc was quenched almost completely after adding Alcian blue 8GX in acidic or weakly acidic media (Figure 3, 2-2’), indicating that Alcian blue 8GX can quench the fluorescence of AlS_4_Pc with high efficiency. Both AlS_4_Pc and Alcian blue have similar large-scale planar structure to phthalocyanine, and thus they form a strong association due to their opposite charges and planar hydrophobic interaction, leading to the formation of a non-fluorescence ion-pair associate (AlS_4_Pc-Alcian blue 8GX) resulting in the quenching of the fluorescence of AlS_4_Pc. Interestingly, the fluorescence of the above system recovered significantly after adding mercuric chloride, and the recovery degree of fluorescence was linearly and positively correlated over a wide range of Hg(II) concentrations.

**Figure 4:**
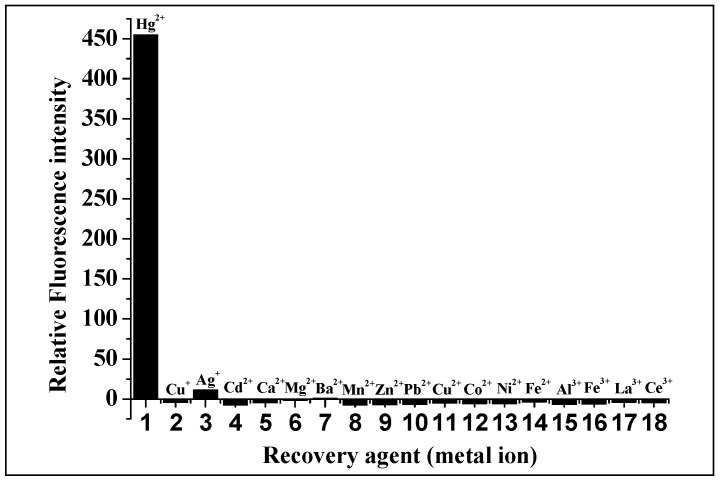
Comparison of recovery effect on the fluorescence of AlS_4_P_c_-Alcian blue 8GX in the presence of different metal ions in an aqueous medium with a pH of 5.0 containing EDTA. The concentration of AlS_4_Pc was 1.0 × 10^−6^ mol/L, Alcian blue 8GX was 1.50 × 10^−6^ mol/L, and EDTANa_2_ was 1.0 × 10^−4^ mol/L. The concentration of all of the metal ions added was 1.0 × 10^−4^ mol/L.

**Figure 5:**
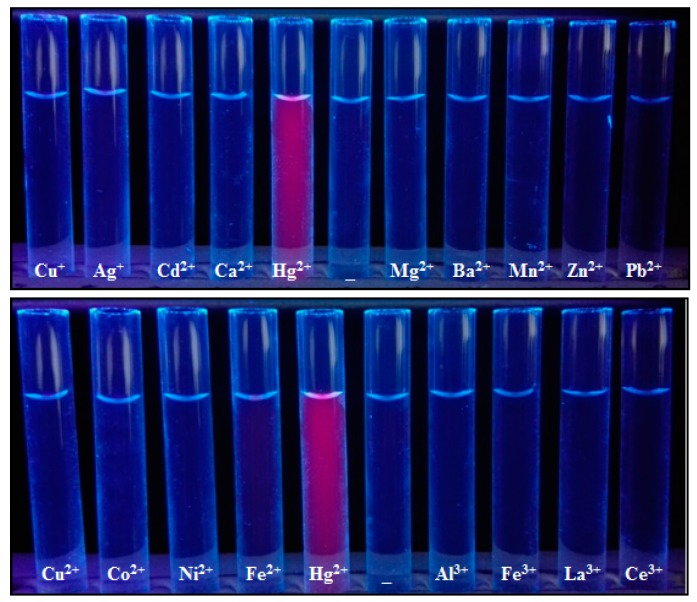
Naked-eye observation of the fluorescence recovery for AlS_4_P_C_-Alcian blue 8GX in the presence of different metal ions including Hg(II) in an aqueous medium. The concentration of AlS_4_Pc was 1.0 × 10^−6^ mol/L, Alcian blue 8GX was 1.50 × 10^−6^ mol/L and EDTANa_2_ was 1.0 × 10^−4^ mol/L. The concentration of all of the metal ions added was 1.0 × 10^−4^ mol/L.

It can be seen from the structure of Alcian blue 8GX (Figure 2) that there are four thioether groups which connect the parent structure of phthalocyanine and the cationic groups on periphery, indicating the presence of sulfur atoms in Alcian blue 8GX. It can be deduced that the strong binding nature of Hg(II) to sulfur atoms (K_sp_ of HgS up to 4 × 10^−53^) makes it easy to react with sulfur atoms in Alcian blue 8GX. This effect remarkably weakens the binding of Alcian blue 8GX to AlS_4_Pc and increases fluorescence due to the release of AlS_4_Pc from the AlS_4_Pc-Alcian blue 8GX associate. This results in a gradual increase in the concentration of free AlS_4_Pc in the solution, and thus the fluorescence obviously recovered with an increase in the Hg(II) (Figure 3, 3~9 and 3’~9’). Further investigations revealed that the presence of the majority of common metal ions could not restore the fluorescence of AlS_4_Pc-Alcian blue 8GX system (Figure 4). It indicates the high specificity for Hg(II) on AlS_4_Pc-Alcian blue 8GX system, which is an important finding from this study. The visualization of mercury ions can also be achieved using this specificity and the red fluorescence emission characteristics of AlS_4_Pc (Figure 5). We believe that the highly selective response of AlS_4_Pc-Alcian blue 8GX to mercury ions lies in the competitive binding effect of Hg(II) with Alcian blue 8GX. Based on the above findings, this study established a new fluorimetric method for the quantitative analysis of Hg(II) with a high selectivity, high sensitivity, and responsiveness in a wide range of concentrations.

### 2.4. Optimization of Experimental Conditions

#### 2.4.1. pH and the Selection of Buffer Medium

The effects of three buffer systems (phosphate buffer of pH 1.0~12.0, B-R buffer of pH 1.0~12.0, and citric acid–disodium hydrogen phosphate buffer of pH 2.0~8.0) on the fluorescence quenching and recovery were investigated. The results showed that the fluorescence recovery ratio (*n* = I_f_/I_f0_) of Hg(II) to AlS_4_Pc-Alcian blue 8GX system was the largest in a buffer solution of pH = 5.0 and the fluorescence quenching ratio [Q%, Q = (I_fT_ − I_f0_)/I_fT_] was close to 100% at a pH of 5.0, so this buffer was finally chosen as the reaction medium.

#### 2.4.2. Selection of Temperature

**Figure 6:**
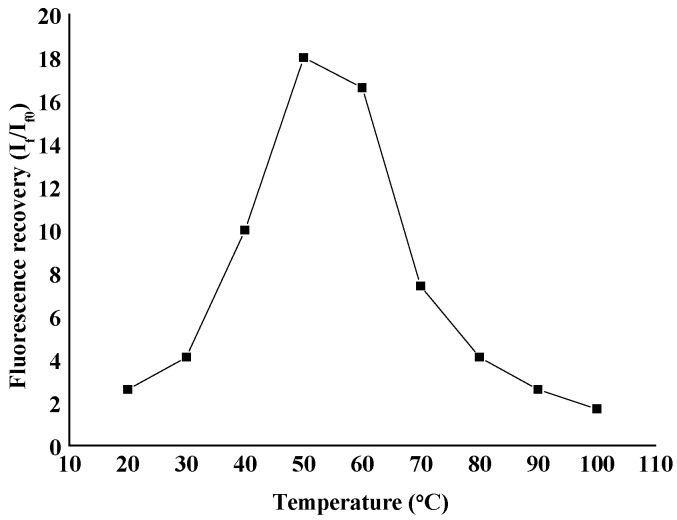
The influence of temperature on the fluorescence recovery of Alcian 8GX—AlS_4_Pc.

The effect of reaction temperature on the fluorescence quenching and recovery of the reaction system was investigated. It was found that the degree of fluorescence quenching dropped with an increase in the temperature, indicating that this quenching system involves a static quenching process (static quenching constant decreases with an increase in temperature). In the range of 20~100 °C, the fluorescence recovery increases at first and then decreases with an increase in temperature. The fluorescence recovery of the system reached the maximum at 50 °C (Figure 6), due to which the reaction temperature was set at 50 °C.

#### 2.4.3. Selection of Reaction Time

**Figure 7:**
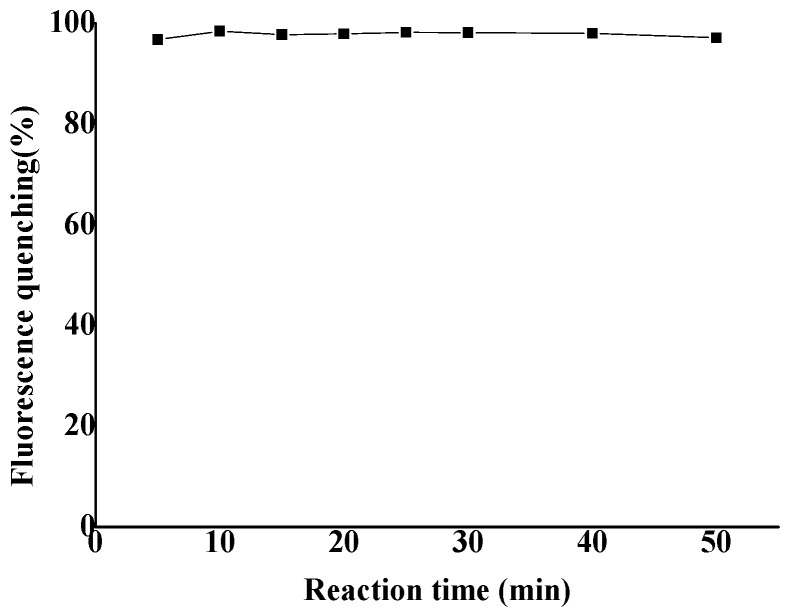
The influence of reaction time on the fluorescence quenching Alcian 8GX on AlS_4_Pc. The concentration of AlS_4_Pc, Alcian blue 8GX, and Hg(II) were 1.0 × 10^−6^ mol/L, 1.50 × 10^−6^ mol/L, and 1.0 × 10^−5^ mol/L, respectively.

The effect of reaction time on the fluorescence quenching and recovery of the system was also investigated. The fluorescence quenching did not show any significant change within 1 h (Figure 7), and the fluorescence recovery increased at first and then decreased with the extending reaction time. The fluorescence recovery of the system reached the maximum after 40 min, which was then chosen as the reaction time. Moreover, after cooling down the reaction for 30 min, both the fluorescence quenching and fluorescence recovery become stable. After 48 h at room temperature, they were basically unchanged, indicating that the reaction system has good stability.

#### 2.4.4. Investigations on the Usage of Alcian Blue 8GX

**Figure 8:**
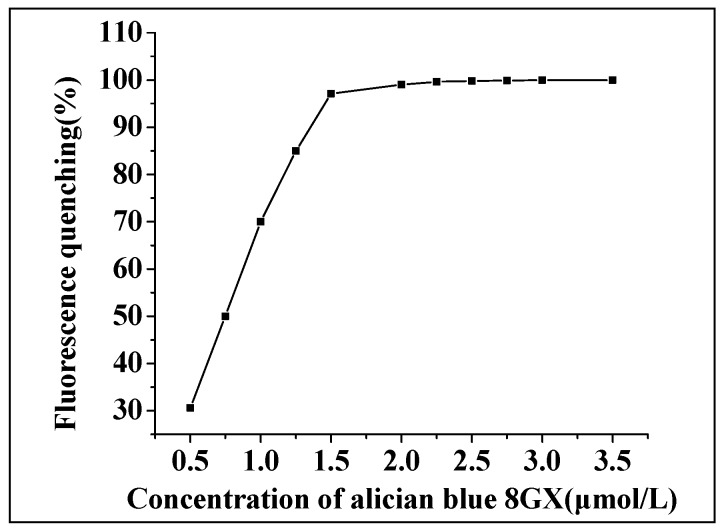
The effect of dosage of Alcian blue 8GX on the degree of fluorescence quenching of the system. The concentrations of AlS_4_Pc and Hg(II) were 1.0 × 10^−6^ mol/L and 1.0 × 10^−5^ mol/L, respectively.

Under the given concentration (1.0 × 10^−6^ mol/L) of AlS_4_Pc and other optimized conditions, the effects of different concentrations of Alcian blue 8GX on the fluorescence quenching (Figure 8) and recovery of the reaction system were investigated. The other conditions remained the same. It was found that as the concentration of Alcian blue increased, the degree of quenching of fluorescence gradually increased until reaching a platform where the quenching value was close to 100%. The results demonstrated that the relative fluorescence intensity of the system increased at first and then decreased with a gradual addition of Alcian blue, and the relative fluorescence intensity reached the maximum when Alcian blue was at 1.50 μmol/L. Therefore, based on the inflection point of the fluorescence quenching curve and the recovery condition of the system, the dosage of Alcian blue was determined to be 1.50 μmol/L.

### 2.5. Effects of Coexisting Substances

Under optimized conditions, the concentration of Hg(II) was set at 2.0 × 10^−6^ mol/L. The interferences of 17 types of common metal ions on the system were investigated and it was found that there were different levels of interference of various metal ions. EDTANa_2_ was added as a masking agent in order to solve this problem, where the concentration of EDTANa_2_ was screened to be of 1.0 × 10^−4^ mol/L. After screening 17 types of metal ions and 9 types of anions, the relative error of all the interfering metal ions was found to be less than ±10% (Table 1). In addition to the interference from I^−^ (I^−^ and Hg(II) may form [HgI_4_]^2−^, which makes Hg(II)unable to bind to Alcian blue 8GX resulting in no recovery in the fluorescence), the interference by the other eight anions (F^−^, Cl^−^, Br^−^, NO_3_^−^, CH_3_COO^−^, SO_4_^2−^, CO_3_^2−^, PO_4_^3−^) was very small, even when the their concentrations were up to 100 times. This indicates that the AlS_4_Pc-Alcian blue 8GX associate has strong anti-interference ability to most of anions and metal ions after adding the masking agent (EDTANa_2_).

### 2.6. Standard Curve

**Figure 9:**
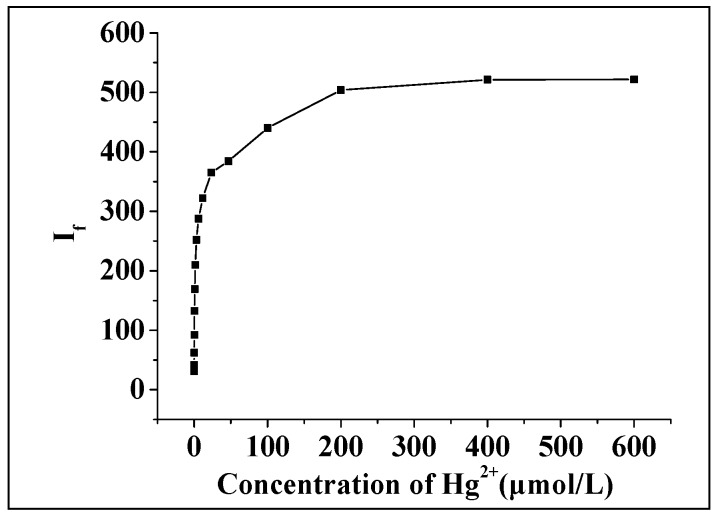
Relationship between fluorescence intensity and the concentration of Hg(II).

**Figure 10:**
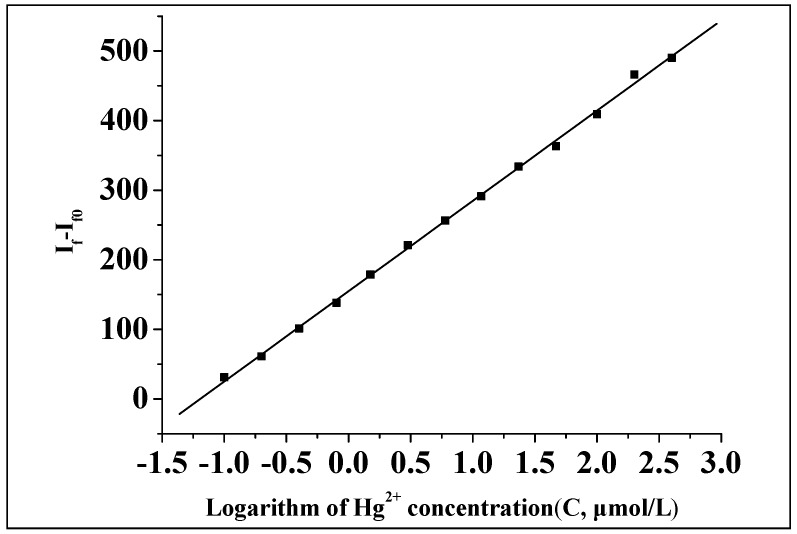
The calibration curve for the determination of Hg(II).

Under the optimal conditions, the fluorescence intensity of the system in the presence of different concentrations of Hg(II) was measured. It was found that the fluorescence intensity at first increased and then remained constant with an increase in the concentration of Hg(II) (Figure 9). A further mathematical analysis showed that the logarithm of the relative fluorescence intensity (ΔI_f_ = I_f_ − I_f0_) to Hg(II) concentration showed a good linearity within a certain order of concentration range (Figure 10). The linear regression equation (calibration curve) was obtained by plotting the logarithmic value (lgC) of the concentration of Hg(II) (C, μmol/L) to the restored relative fluorescence intensity (ΔI_f_ = I_f_ − If_0_), which showed ΔI_f_ = 154.82 + 129.71lgC, where r = 0.9994, the linear range was 0.1–400 μmol/L, and the detection limit was 0.0104 μmol/L.

### 2.7. Analysis of Practical Samples

The prepared fluorescent probe, AlS_4_Pc-Alcian blue 8GX, was used to determine the content of three types of mineral water, where the mercury ion was not detected. Subsequently, the standard solutions of different concentrations of Hg(II) were added to the above mineral water, followed by the performance of determination described in 4.2. For example, 1 mL of Lungful mineral water and 30 μL of Hg(II) solution (1.0 × 10^−4^ moL/L) was added in sequence to a 10 mL glass tube containing reaction reagents, followed by dilution to get a final volume of 3.0 mL. The mixed solution containing Hg(II) with a concentration of 1.0 μmoL/L was measured by a standard procedure, and the recovery was calculated (112.5%). The results obtained from these studies have been shown in Table 2. From these results, it could be noted that the recoveries of Hg(II) in three types of mineral water were satisfactory when the scalar quantity (e.g., the concentration of Hg(II) added is 0.1 µmol/L) was low, so the fluorescent probe could be used for the determination of Hg(II) content in simple real samples at present.

## 3. Materials and Methods

### 3.1. Materials

Tetrasulfoaluminium phthalocyanine (AlS_4_Pc) was purchased from J&K Technology Co., Ltd. (Beijing, China) and stored at 4 °C in the dark at a concentration of 1.0 × 10^−2^ mol/L. Alcian blue 8GX was supplied by Bioengineering Co., Ltd. (Shanghai, China) and stored at a concentration of 1.0 × 10^−2^ mol/L, and diluted to 1.0 × 10^−4^ mol/L before use; Disodium ethylenediaminetetraacetate (Wuhan Chemical Plant); mercuric chloride (Tonghuo Mercury Reagent Factory, Guizhou, China) were utilized as obtained; pH buffer solutions in a wide range (mixed with hydrochloric acid, dipotassium hydrogen phosphate, potassium dihydrogen phosphate, and sodium phosphate); Britton–Robinson (B-R) buffer (mixed preparation with phosphoric acid, acetic acid, boric acid, and sodium hydroxide) and disodium hydrogen phosphate–citrate buffer were used. All other reagents are of analytical grade and the water used was of high purity.

### 3.2.Experimental Methods

The following solutions were prepared in a beaker: 30 μL of AlS_4_Pc (1.0 × 10^−4^ mol/L), 45.0 μL of Alcian blue 8GX (1.0 × 10^−4^ mol/L), 30.0 μL of EDTANa_2_ (1.0 × 10^−2^ mol/L), and 300.0 μL of buffer with pH = 5.0. By adding water, 405 μL of the above mixture and Hg(II)solution to a 10.0 mL glass tube, the final volume was made to 3.0 mL, and mixed thoroughly using a vortex mixer. The solution was then heated in a water bath at 50 °C for 40 min and then cooled for 30 min before subjecting it to the measurements of fluorescence. The excitation wavelength was at 613 nm and the excitation slit was 5.0 nm, whereas the emission slit was 4.5 nm. The fluorescence intensity of AlS_4_P_C_ tube without Alcian blue 8GX and Hg(II) was recorded as I_fT_. The blank tube with intensity recorded as I_f0_ without Hg(II). The fluorescence intensity of sample tubes with Hg(II) was recorded as I_f_. The fluorescence quenching ratio (Q%) was then calculated by, Q = (I_fT_ − I_f0_)/I_fT_; whereas the fluorescence recovery factor (n) was calculated by, n = I_f_/I_f0_; and the difference in the fluorescence intensity before and after recovery could be expressed by ΔI_f_ = I_f_ − I_f0_.

## 4. Conclusions

In summary, the AlS_4_Pc-Alcian blue 8GX ion-pair fluorescence probe with low fluorescence background was constructed based on the high-efficiency fluorescence quenching effect of Alcian Blue 8GX on AlS_4_Pc. The screening experiments for common metal ions revealed that only Hg(II) could significantly restore the fluorescence of AlS_4_Pc-Alcian blue 8GX, indicating that AlS_4_Pc-Alcian blue 8GX probe has high specificity for the recognition of Hg(II). Inspired by these results, a new fluorimetric method has been established with high specificity and high sensitivity for the quantitative analysis of Hg(II), and its response range is found to be more than three orders of magnitude. It confirms that AlS_4_Pc-Alcian blue 8GX ion associate is promising as an excellent red fluorescent probe which further opens up new applications of red-emitting fluorescent probes based on functional phthalocyanine compounds. What is more worth mentioning is this study has proposed a novel strategy for constructing new fluorescent probes through molecular assembly. By the association of structure-matched molecules, novel fluorescent probes could be achieved with high specificity to a specific species. Compared with ‘turn-on’ fluorophores reported in literature, compounds used in this study for constructing the associate are commercially available, thus opening an easy way to achieve fluorescent probes without cumbersome organic synthesis. Besides, the constructed probe is fully water-soluble. It can be expected that a series of functional phthalocyanine based fluorescent probes would be developed according to this principle.

## Figures and Tables

**Table 1:** Effect of 17 metal ions on the determination of Hg(II).

Metal Ions	Concentration (mol/L)	Relative Error/%	Metal Ions	Concentration (mol/L)	Relative Error/%
Ag^+^	2.0 × 10^−5^	+8.71	Fe^3+^	1.0 × 10^−5^	−7.99
Cu^2+^	4.0 × 10^−5^	−7.42	Al^3+^	1.5 × 10^−5^	−7.53
Cd^2+^	1.0 × 10^−5^	−9.33	Ce^3+^	1.0 × 10^−5^	−9.25
Ca^2+^	2.0 × 10^−5^	−7.11	La^3+^	1.0 × 10^−5^	−8.55
Ba^2+^	8.0 × 10^−5^	−5.65	F^−^	1.0 × 10^−5^	−7.25
Mg^2+^	6.0 × 10^−5^	−8.84	Cl^−^	2.0 × 10^−4^	−4.64
Co^2+^	1.0 × 10^−5^	−9.45	Br^−^	2.0 × 10^−5^	−6.75
Ni^2+^	1.0 × 10^−5^	−9.01	I^−^	2.0 × 10^−6^	−40.37
Cu^2+^	2.0 × 10^−5^	−8.84	N0_3_^−^	2.0 × 10^−4^	−1.71
Zn^2+^	1.0 × 10^−5^	−9.31	SO_4_^2−^	2.0 × 10^−4^	−2.53
Mn^2+^	1.0 × 10^−5^	−9.01	CO_3_^2−^	2.0 × 10^−5^	7.17
Pb^2+^	2.0 × 10^−5^	−8.52	PO_4_^3−^	2.0 × 10^−4^	5.06
Fe^2+^	1.5 × 10^−5^	−5.81	CH_3_COO^−^	2.0 × 10^−4^	0.84

([Hg(II)] = 2.0 × 10^−6^ mol/L).

**Table 2:** Determination of Hg(II) in three mineral water samples by the present method.

Samples		Hg(II) Added (µmol/L)	Hg(II) Detected (µmol/L)	Recovery (%)
Lungful Shanquan (pH 6.89)	1	0	Not detected	_
	2	0.1	0.107 ^a^ ± 0.12 ^b^	107.0
	3	1.0	1.125 ^a^ ± 0.23 ^b^	112.5
Baisuishan (pH 6.86)	1	0	Not detected	_
	2	0.1	0.108 ^a^ ± 0.04 ^b^	108.0
	3	1.0	1.201 ^a^ ± 0.21 ^b^	120.1
Biaozhi (pH 7.03)	1	0	Not detected	_
	2	0.1	0.104 ^a^ ± 0.05 ^b^	104.0
	3	1.0	0.999 ^a^ ± 0.13 ^b^	99.9

^a^ average of three results, ^b^ standard deviation.
